# The Ecological Importance of Toxicity: Sea Anemones Maintain Toxic Defence When Bleached

**DOI:** 10.3390/toxins11050266

**Published:** 2019-05-11

**Authors:** Cassie M. Hoepner, Catherine A. Abbott, Karen Burke da Silva

**Affiliations:** College of Science and Engineering, Flinders University, Adelaide, SA 5001, Australia; cassie.hoepner@flinders.edu.au (C.M.H.); cathy.abbott@flinders.edu.au (C.A.A.)

**Keywords:** anemone, bleaching, climate change, haemolysis, resilience, venom

## Abstract

Cnidarians are amongst the most venomous animals on the planet. They are also under significant threat due to the impacts of climate change. Corals and anemones undergo climate-induced bleaching during extreme environmental conditions, where a loss of symbiotic photosynthetic algae (zooxanthellae) causes whitening in colour, loss of internal food supply, and reduction in health, which can ultimately lead to death. What has yet to be determined is whether bleaching causes a reduction in the production or quality of venom. In this study, the sea anemone *Entacmaea quadricolor* was exposed to long-term light-induced bleaching to examine the effect that bleaching has on venom. Venom quality and quantity, as determined through lethality and haemolysis measures and nematocyst production was highly preserved over the five-month imposed bleaching event. Maintenance of venom and nematocyst production, despite a loss of an internal food source provided by endosymbiotic algae, indicates both the ecological importance of maintaining toxicity and a remarkable resilience that anemones have to major environmental stressors.

## 1. Introduction

Cnidarians, a large group of marine invertebrates, are comprised of approximately 9400 species [1] and are amongst some of the most venomous animals on the planet [2,3]. Cnidarian venom is composed of a variety of active compounds that effect voltage gated Na^+^ and K^+^ channels [4], many of which have been tested for use as human therapeutic drugs [5,6]. Sea anemone venom contains multiple biologically complex active substances including neurotoxins and cytolytic proteins and peptides [4]. The two most commonly studied anemone toxins are the 2–5 kDa neurotoxins that bind to voltage-gated sodium (Na_v_) and potassium (K_v_) channels as well as 15–20 kDa cytolysins [3,4,7]. Cytolysins can be classified into four polypeptide groups [4] and function by forming pores in cell membranes which promote osmotic imbalance and cell lysis. These toxins are highly potent and largely unstable [7]. Low molecular weight neurotoxins, in contrast, induce cardiotoxic effects, such as arrhythmias, from the inactivation of the Na_v_ channel, and cause cardiac arrest due to calcium ion overloading [3].

Anemone venom is delivered from specialised stinging cells called nematocysts, [3,4] that are fired semi-autonomously [8] after mechanical or chemical stimulation and can penetrate skin to deliver venom. Nematocysts are utilised by anemones for a range of functions, including protection from predators, interspecific competition, and the acquisition of prey [8,9]. In addition to nematocysts, anemones also secrete a mucus coat as a form of self-protection that contains cytolysins and neurotoxins [10]. Toxins aid in a sessile lifestyle [11], by enabling anemones to target mobile prey such as fish and crustaceans [3,4,10]. Anemones are capable of paralysing both predators and prey through the injection of toxins via nematocysts, causing pain, loss of muscle control, and tissue damage [10]. Cytolysins, even when diluted (<0.5 µg mL^−1^), have been shown to cause mortality in fish within an hour, because of impaired gill function [4,10]. Venom toxicity is a vital aspect of the anemone’s ability to thrive within their ecological niche. The diverse range of toxins and configurations characterised to date indicate the strong selection pressure maintained during toxin evolution and the need for anemones to adapt to changes within their environment [11]. 

Research into anemone venom to date has focused almost exclusively on the molecular make-up and protein function of venom, with very little investigation into ecological function [3,4]. In particular there is a large gap surrounding how venom responds to changes in environmental conditions. Anemones are a vital component of reef ecosystems and similar to corals are vulnerable to the effects of climate change, as they have a symbiotic relationship with the same photosynthetic alga, zooxanthellae that is sensitive to anthropogenic effects [12,13]. Numerous environmental stressors, including elevated sea surface temperature, high visible light and ultraviolet radiation, changes in salinity, increased sedimentation, or the accumulation of pollutants or nutrients can all cause a breakdown in the relationship between hosts (corals and anemones) and algal symbionts resulting in bleaching [14,15,16,17,18]. Bleaching causes a deterioration of body condition with subsequent loss of colour due to the expulsion of zooxanthellae and photosynthetic pigments [19,20]. Studies by Saenz-Agudelo, et al. [21] and Hobbs, et al. [22] demonstrate a negative effect of bleaching in relation to anemone size, with a significant reduction (up to 74%) occurring throughout the bleaching period.

Production of venom and firing of nematocysts requires a significant investment of energy [23]. The maintenance of venom supplies during extended stress events and the energy costs associated with bleaching, begs the question of whether anemones have the capacity to continue making venom and its delivery system, required for protection and feeding under these circumstances. As bleaching occurs energy input from algal symbionts (normally up to 85% [24]) decreases. Energy requirements normally utilised for venom synthesis are potentially transferred to maintain daily metabolic requirements [21], leaving anemones in a compromised condition.

The anemone *Entacmaea quadricolor* is a common, geographically widespread species [25] that is also host to symbiotic anemonefish [8,26]. The venom of *E. quadricolor* contains the high potency group ΙΙ peptide (15–20 kDa cytolysins) [4], common to many host anemones, as well as type ΙΙΙ neurotoxic compounds (2–5 kDa) [3]. Within Australia, *E. quadricolor* is found along the northeastern coast from Far North Queensland to North Solitary Island, New South Wales [27] and has a thermal tolerance threshold between 25 and 27 °C [26]. Average summer temperatures in this region are currently hovering around 26 °C, thus, this species is at risk in Australian waters, as it is within 1 °C of its thermal threshold [26,28]. Pontasch, et al. [29] predicted a 2 °C raise in temperature for this region by 2050 that could be highly detrimental to this species, with excess bleaching occurring and uncertainty regarding its ability to acclimate. How the impact of anthropogenic disturbance and consequential bleaching in anemones affects venom synthesis and nematocyst production is not known. This study aims to quantify the effect of bleaching over time on venom and nematocyst production in the anemone *E. quadricolor* under laboratory conditions.

## 2. Results

### 2.1. Anemone Bleaching

**Figure 1:**
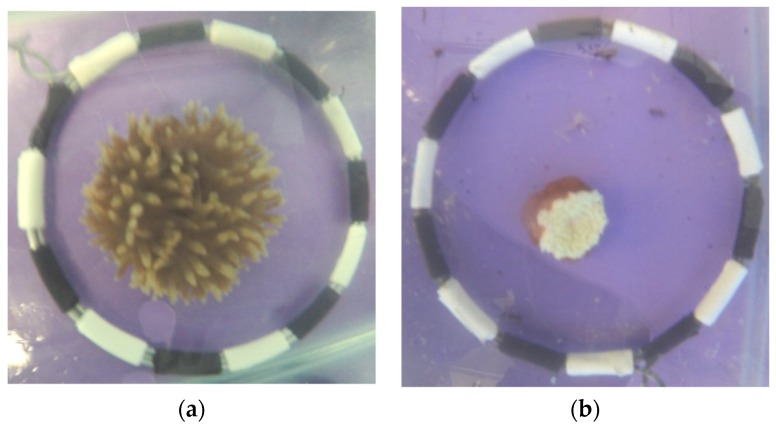
Changes in anemone size and colour due to bleaching: (**a**) anemone at week 0, pre- bleaching; and (**b**) week 18 after being exposed to light deprivation for a period of five months.

Significant bleaching occurred in all anemones in both light treatments, as indicated by a significant increase in percentage whiteness (Figure 1). A significant decrease in visible tentacle area over the same bleaching time period was also observed [30] (Figure 1). During week 15 one individual anemone from the FLURO group died (1/18, 5% mortality), while all other anemones from both groups survived the entire five-month bleaching process. 

### 2.2. Venom Characterisation 

**Figure 2:**
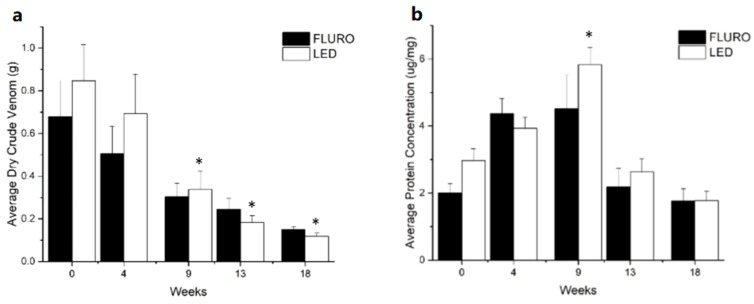
Differences in venom collected with bleaching overtime (mean ± SE): (**a**) weight of dry crude venom (g); and (**b**) protein concentration (µg/mg) of dry crude venom. LED (n = 9) FLURO (n = 8). * indicates weeks significantly different to week 0.

The volume of dry crude venom collected during milking decreased over the five-month bleaching period. By week 18 average crude venom dry weight produced by anemones from both the LED and FLURO groups decreased by 68.1% (± 10.9 SE) for the FLURO group and 81.7% (± 3.4 SE) for the LED group (Figure 2a). From week 9 onwards the average weight of dry crude venom was significantly lower than the volume produced during week 0 for both the LED and FLURO groups (FLURO F(1.561, 12.486) = 10.604, *p* < 0.003. LED F(1.763, 12.339) = 4.305, *p* < 0.042). 

Protein concentration was used throughout the study as a refined indicator of venom toxicity [1,27]; as the amount of dry crude venom collected from the anemones was not representative of the resultant protein concentration (Figure 2). The protein concentration of the venom was found to be significantly different over time (FLURO F(4,16) = 13.987, *p* < 0.0005. LED F(4,16) = 6.629, *p* < 0.002) (Figure 2b). Venom protein concentration peaked in week 9 being almost double what it was in week 0; protein concentrations subsequently returned to initial concentration levels in weeks 13 and 18. 

**Figure 3:**
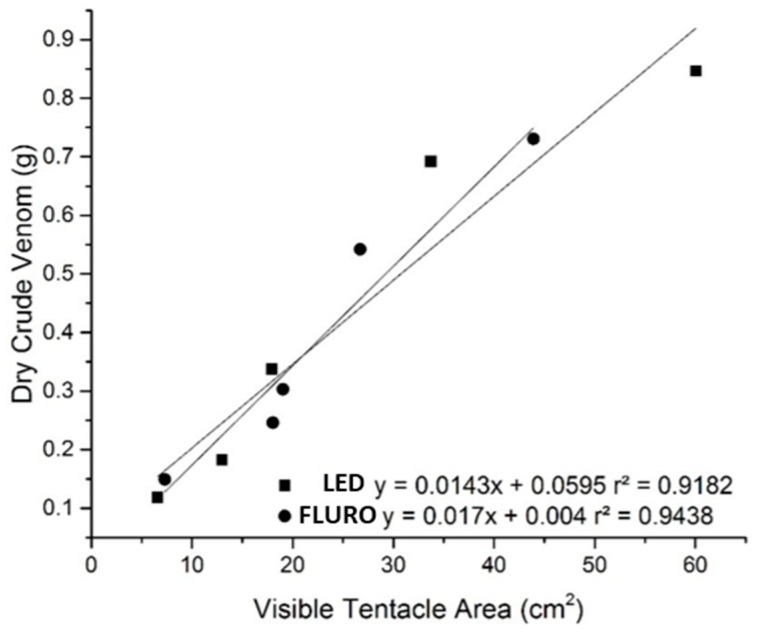
Correlation between average dry crude venom collected (g) and average visible tentacle area (cm^2^). LED (n = 9) FLURO (n = 8).

Visible tentacle area strongly correlated with the amount of dry crude venom, showing that as body size decreases in response to bleaching, less venom was produced (FLURO r^2^ = 0.94, *p* < 0.006. LED r^2^ = 0.92, *p* < 0.01) (Figure 3). No correlation was found between visible tentacle area and protein concentration of the dry crude venom. 

### 2.3. Protein Composition of Venom 

**Figure 4:**
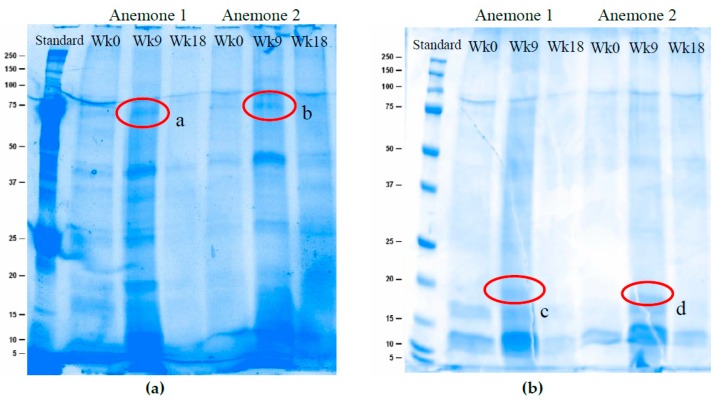
Venom samples contain multiple proteins and peptides which differ in intensity over time with bleaching. Ten µg of protein from the reconstituted venom samples was loaded onto a 12% SDS-PAGE, two biological replicates (Anemone 1 and Anemone 2) from week 0, 9, and 18 were compared. (**a**) BioRad stain-free fluorescence protocol which highlights tryptophan containing peptides and proteins; and (**b**) BioRad Coomassie Brilliant Blue R-250 staining. Bands of interest are circled in red and labelled a–d.

SDS-PAGE analysis showed that as bleaching occurred the expression of prominent protein bands changed. Both the stain-free fluorescence and Coomassie Brilliant Blue R-250 staining showed that the protein composition of the anemone venom samples is complex with >20 bands observed using both methods. The stain-free fluorescence protocol allowed visualization of greater complexity across all size ranges (Figure 4a) where as the Coomassie Brilliant Blue R-250 staining showed more distinct bands in size range of known toxins (2–20 kDa) (Figure 4b). Across both images the protein composition from week 9 was found to be the most different compared to the other weeks. In particular, highlighted bands a and b occur at approximately 75 kDa and the intensity (adjusted volume) increased by 38.5% for band a and 93.1% for band b in week 9 compared to week 0, before disappearing completely in week 18 (Table S1). Consistently, bands were observed at 11 kDa and 16 kDa across all weeks. However, these bands also vary in intensity throughout the bleaching process, with some increasing and others decreasing (Table S1). Highlighted bands c and d depict bands at 18 kDa that appear only in week 9.

### 2.4. Lethality and Haemolysis 

**Figure 5:**
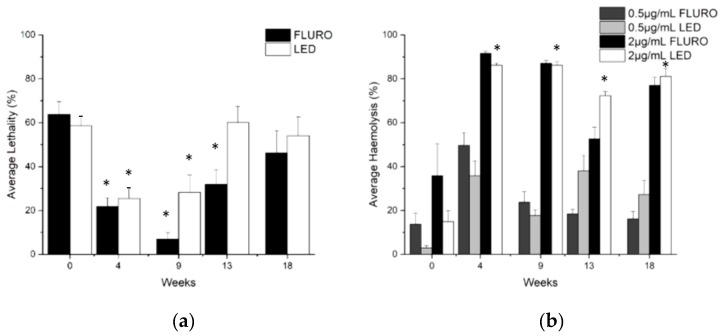
Anemone venom lethality and lysis changes with bleaching overtime (mean ± SE) (**a**) *Artemia* lethality (%) at fixed 4 µg mL^−1^ protein concentration; and (**b**) haemolysis (%) at protein concentrations of 2 and 0.5 µg mL^−1^. LED (n = 9) FLURO (n = 8). * indicates weeks significantly different to week 0.

Initial experiments with week 0 venom determined that at a protein concentration of 4 µg/mL reconstituted venom led to approximately 50% *Artemia* lethality after 24 h (Figure S1). As anemones bleached, lethality varied significantly at this protein concentration (FLURO F(4,32) = 14.889, *p* < 0.0005. LED F(4,32) = 6.034, *p* < 0.001) (Figure 5a). Venom lethality significantly decreased for both groups in weeks 4 and 9 (and week 13 for the FLURO group only) with between 49.6–88.2% (±5.6–11.9 SE) (FLURO group) and 52.9%–58.4% (±5.7–12.5 SE) (LED group) fewer *Artemia* killed by venom collected these weeks. Lethality returned to original levels by week 18 for both treatments. 

Initial experiments with week 0 venom determined that at a protein concentration of 2 µg/mL reconstituted venom led to approximately 50% haemolysis (Figure S2). As bleaching occurred, there was a significant increase in haemolysis at this concentration (FLURO F(1.126,4.502) = 14.961, *p* < 0.014. LED F(4,16) = 96.950, *p* < 0.0005) with >75% more lysis produced when the assay was performed on samples from weeks 4-18. Therefore, a lower dose of 0.5 µg/mL (1/4 concentration) was also tested for comparison (Figure 5b). There was again a significant change in percentage lysis caused by venom once bleaching occurred, using this lower concentration (FLURO F(4,16) = 7.914, *p* < 0.001. LED F(4,16) = 5.040, *p* < 0.008). 

### 2.5. Nematocysts 

**Figure 6:**
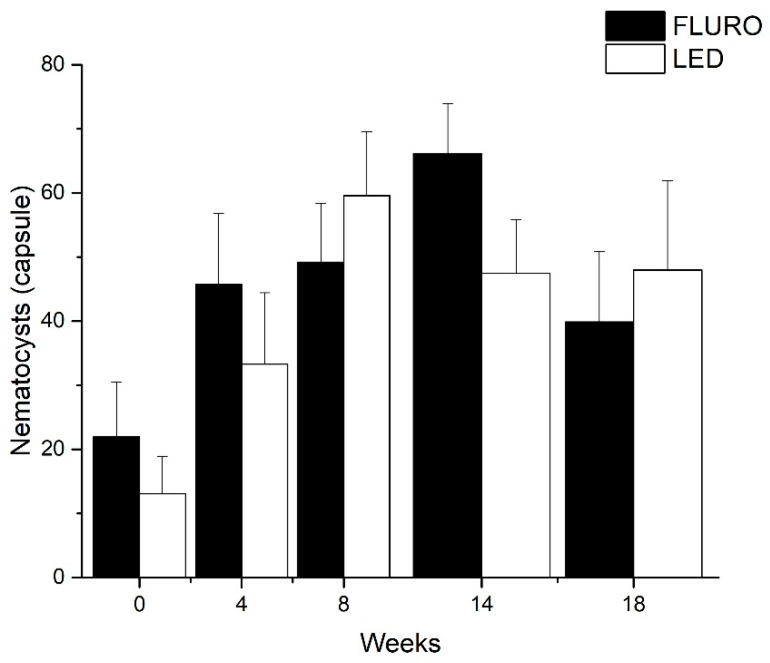
Total nematocyst count changes during bleaching event (mean ± SE). LED (n = 9) FLURO (n = 8).

Nematocyst production changed significantly throughout the bleaching process (FLURO F(4,28) = 2.836, *p* < 0.043. LED F(4,32) = 3.743, *p* < 0.013) (Figure 6). As bleaching occurred anemones on average had an increase of between 5.7–12.1% (± 2.5–9.0) (FLURO) and 14.8–61.4% (± 12.7–46.9 SE) (LED) in the number of nematocysts produced in weeks 8–18 compared to week 0.

## 3. Discussion

The negative effects of climate change are impacting ecosystems faster than ever before [24,31]. How climate change influences the production and deployment of animal venom has received little investigation even though it is vital for the growth and survival of various toxin producing species [4,8]. Despite an imposed high physiological challenge, in the form of two different light stressors, used to initiate and maintain bleaching in this study, only a single anemone died (1/18, 5% mortality). The remaining anemones continued to produce viable venom that was able to elicit a lethal and haemolytic response. As anemones bleached and lost their photosynthetic algae, they decreased in size but continued to produce venom, although at a reduced volume, suggesting a trade-off between venom production and investment in growth. This finding suggests the high importance of venom production for anemone survival. Hydrocorals have also been found to maintain toxicological activity when bleached [32] suggesting a strong ecological advantage to maintaining venom production. Interestingly, corals when bleached have a much higher mortality rate than anemones, as they are highly susceptible to viral and bacterial invasion in a bleached condition [33,34,35]. Our study demonstrates for the first time the resilience of *E. quadricolor* anemones during an extended bleaching event, as demonstrated by their survival and maintenance of vital protective toxins. The ability of *E. quadricolor* to maintain function and synthesis of toxins and nematocysts, despite the loss of their endosymbionts, will help them survive during challenging environmental conditions caused by climate change. 

Up to 85% of the anemone nutritional budget is provided by their *Symbiodinium* symbionts [14,24]. Therefore, the loss of endosymbionts following a bleaching event results in the loss of essential nutrient provision (e.g., carbon and nitrogen [36]). During this time, anemones underwent a negative growth period, similar to what has been found during times of high stress or starvation [30,37], by consuming their own fatty acid reserves [38]. This mechanism of negative growth is likely responsible for enabling the maintenance of anemone toxicity and ultimately survival until the acquisition of new symbiotic algae.

Haemolysis reacted differently to lethality throughout the bleaching event. At both protein concentrations tested (2 µg mL^−1^ and 0.5 µg mL^−1^) haemolysis increased but did not return to pre-bleaching levels. Venom samples collected from bleached anemones in this study were significantly more potent than pre-bleaching samples, suggesting investment in additional protection during stress events. Similarly, haemolysis on ethanol extracts of bleached hydrocoral fragments were found to be slightly more potent than control fragments [32]. Nematocyst production was also found to increase significantly in response to bleaching, again showing a positive protective mechanism. An increase in both haemolytic activity and nematocyst production could aid anemone survival during high stress events, such as bleaching. This would enable anemones to reduce reliance on autotrophic carbon stores for food, as their endosymbionts are no longer providing them with essential nutrients [21], and focus on external collection of resources via the capture of prey. 

The increase in protein concentration found in week 9 indicates the expression of additional proteins within the venom sample. Sencic and Macek [39] found that the protein content of anemone venom remains relatively stable between monthly sampling, suggesting that the changes in protein concentration found in this study were likely caused by anemone response to bleaching conditions. Other organisms have also been found to show changes in the expression of certain proteins when in altered environmental conditions. For example, Pacific oysters (*Crassostrea gigas*) and King George whiting (*Sillaginodes punctata)* increase expression of heat stress proteins during extended stress events [40,41,42]. 

Heat stress proteins (HSPs) are produced by organisms as a coping mechanism to reduce the damaging effects caused by changes in environmental conditions [43,44]. During extended stress events, such as those that cause bleaching, the expression of HSPs can increase [40,42]. Expression of HSP 60 and HSP 70 associated with thermo-tolerance and temperature rise have been found in anemones [44,45] as have other low molecular weight HSPs (28–29 kDa) [44]. The expression of other non-toxic proteins could explain the results of the *Artemia* lethality experiment. *Artemia* lethality did not increase with increasing protein concentration as the expression of other proteins may have diluted the toxin present in the venom, resulting in a lower number of *Artemia* killed. 

The protein concentration of anemone venom returned to initial levels after a peak in week 9. This indicates a possible acclimation to the stressors the anemones were experiencing. The additional proteins produced during week 9 may no longer have been needed, thus the concentration of toxin proteins in the crude venom (as indicated by the number of *Artemia* killed), returned to pre-bleaching levels. A study by Cubillos, et al. [46] found that anemones were able to acclimate cellularly to UV induced stressors by increasing mycosporine-like amino-acids (<1 kDa). The presence of HSPs in the venom of anemones has yet to be explored, however the intensity of a band approximately 75 kDa in size increased in week 9 which may represent anemone HSP. Fractionation and purification of the dry crude venom may enable the identification of different proteins expressed throughout the bleaching process to be ascertained, as well as their associated function in helping to maintain anemone survival. 

## 4. Conclusions 

*E. quadricolor* and other host anemones play an essential role in reef ecosystems through their three-way symbiosis with anemonefish and photosynthetic algae. The ability of anemones to maintain high levels of both nematocyst and venom production during times of high stress will aid in their survival and consequently the survival of dependent anemonefish. The resilience anemones demonstrated to bleaching events throughout this study is a positive indicator for future anemone survival in the wild under changing environmental conditions. The anemones ability to maintain venom production while subjected to environmental changes is particularly important given their role in coral reef ecosystems more broadly. 

## 5. Materials and Methods 

### 5.1. Bleaching of Anemones 

Eighteen healthy *E. quadricolor* anemones were purchased from the aquarium livestock trader Cairns Marine©, Queensland, Australia, and transported by air to the Animal House facility at Flinders University, South Australia. The initial milking was performed two days after anemones arrived at the Animal House to allow time for acclimation to aquaria and for pre-bleaching assessment. Bleaching was induced gradually over a period of five months via light deprivation. Each anemone was placed in individual aquaria (30 L) (one individual per tank). Anemones were randomly assigned to two groups; LED lighting (Aqua One MariGlo LED 90 (80–400 µmol m^−2^s^−1^, 1385–3461 lux) (n = 9) and fluorescent lighting (ambient room lights 1–8 µmol m^−2^s^−1^, 66–265 lux) (n = 9) (see Figure S3 for wavelengths of each light). Both light sources were on a 12-h day night cycle (8:00–20:00 h) and each aquarium was linked to a larger recirculating 24-tank system that included six tanks each with a pair of anemonefish (*Amphiprion ocellaris*) present for nutrient cycling. Seawater was filtered from a sump to ensure all 24 linked tanks maintained a controlled and constant environment (25 °C ± 2, salinity 33 ± 2, pH 8.3 ± 0.2). Anemones and water quality were monitored daily, and food was provided to the anemones in the form of a small piece of whitebait on a weekly basis. 

### 5.2. Venom Collection 

Venom was extracted using a milking technique as previously described [39]. This is a sustainable method of venom collection allowing for repeated milking, rather than using an animal homogenate. The venom was extracted by gently massaging the tentacles of the anemone inside a plastic aquarium bag. Each individual was milked once a month for five months, so as not to reduce the toxicological quality of the extracted venom [39].

### 5.3. Venom Characterisation 

Crude venom samples were frozen gradually (−20 °C then −80 °C), lyophilised (Christ Beta 2–8 Freeze Dryer, Osterode am Harz, Germany) and stored at −80 °C until needed for the assays. The protein content of the dry crude venom was determined before each toxicology assay using the Pierce^TM^ Bicinchoninic Acid (BCA) protein assay kit (Thermoscientific, Carlsbad, CA, USA) as previously described [47]. Estimates of protein content of crude venom was obtained using a bovine serum albumin (BSA) standard curve. Visible tentacle area was measured using ImageJ 1.50i (open source) [48] via methods adapted from Chow, et al. [49]. The diameter of the reference circle (10.5 cm) (Figure 1) was used to standardise the photo and the full area of tentacle expansion of the anemone was measured using the freehand tool [30].

### 5.4. Electrophoresis 

Sodium dodecyl sulphate polyacrylamide gel electrophoresis (SDS-PAGE) of venom samples was performed as previously described [50]. Venom samples containing 10 µg protein were combined with an equal volume Leammli 2× sample buffer and ran on a 12% Mini-PROTEAN® TGX Stain-Free™ Protein Gel (BioRad, Hercules, CA, USA) at 200 V for 40 mins using a 10× Tris Glycine SDS running buffer (pH. 8.3). Molecular masses were determined using 5 µL of Precision Plus Protein Dual Xtra Standard (BioRad). The gel was first visualised stain-free via fluorescence. The gel was then stained using a Coomassie Brilliant Blue R 250 stain (BioRad 161-0786) for 24 h and allowed to de-stain for 72-h using 10% acetic acid and 10% methanol in water before visualisation.

### 5.5. Artemia Lethality Assay 

Venom lethality was tested using the standardised *Artemia* nauplii toxicity test (ARC-Test) as previously described [51]. *Artemia franciscana* cysts were obtained from the *Artemia* Reference Centre, Ghent University, Ghent, Belgium (www.aquaculture.ugent.be) and hatched according to strict guidelines [51]. Autoclaved artificial seawater (Instant Ocean, Blacksburg, VA, USA: salinity 35.2, pH 8.1) was used for both hatching artemia and in the assay itself. Ten brine shrimp nauplii were exposed to a range of anemone venom protein concentrations (0.5, 1, 1.5, 2, 3, 5 µg mL^−1^) overnight. From this, the concentration of 4 µg mL^−1^ was chosen to test for changes in lethality of the venom overtime (Figure S1). Reference and negative control tests were carried out using potassium dichromate (Sigma Aldrich, Castle Hill, NSW, Australia) (LC_50_ 0.063 µg mL^−1^) and milli-Q water, respectively, in the place of venom. 

### 5.6. Haemolysis Assay 

The cytolytic properties of anemone venom can be easily quantified using haemolysis measurements as previously described [52]. The haemolytic assay was performed using sheep blood (CPDA-1/ACD) obtained from Applied Biological Products Management (Adelaide, South Australia, Australia) and stored at 4 °C. A series of crude venom dilutions (0.1, 0.5, 1, 2, 3, 4 µg mL^−1^ protein) were applied to the erythrocyte suspension in a 96-well plate. The suspension was centrifuged at 1500 *g* (Hermle Z400K, Wehingen, Germany) and the absorbance of the supernatant was measured at 540 nm (Fluostar Omega 96-well plate reader, BMG Labtech, Offenburg, Germany) to test for the release of haemoglobin. From this the concentrations of 2 and 0.5 µg mL^−1^ was chosen to test for changes in haemolysis as a consequence of bleaching over time (Figure S2). A positive (2% Triton X-100, Sigma Aldrich) and a negative (phosphate buffered saline) control were used in each run of the assay [52]. 

### 5.7. Nematocyst Sampling and Quantification 

Nematocysts were sampled fortnightly for the first eight weeks of the study and then were sampled twice more over the following two months to reduce impact on the shrinking individuals. Nematocysts were sampled using a prepared microscope slide with fresh prawn residue rubbed on it. The dried slide was pulled across the anemone tentacles to induce firing of the nematocysts [23]. Slides containing nematocysts were stained using Methylene Blue (Sigma Aldrich) and nematocysts were quantified using a compound microscope (Olympus CH-2, Notting Hill, VIC, Australia) at 40× magnification to count the number of capsules present. The number of capsules in the field of view were counted across eight consistent replicates per slide. 

### 5.8. Analysis 

Repeated measures analysis of variance (RM-ANOVA) in SPSS 2015 (IMB Corp., Armonk, NY, USA) were used to test for changes in dry crude venom, protein concentration, lethality, haemolysis, and nematocyst production overtime. Mauchly’s test of sphericity was tested for before the use of the RM-ANOVA and a greenhouse geisser correction was applied when sphericity was not met. Post hoc tests with Bonferroni adjustments were used to identify significant differences (*p* < 0.05). The RM-ANOVA is reported in text as (Treatment F(^df^time, ^df^error) = F-value, *p*-value). The *Artemia* lethality and haemolytic assays were analysed using best fit dose response curves to determine the protein concentration of venom to achieve approximately 50% lethality or lysis. Correlations between variables were tested for using Pearson’s Correlation. All graphs were created in Origin 2015 (OriginLab, Northampton, UK). Quantfication of bands from the gel electrophoresis was performed in Image Lab 6.0 (BioRad). Lanes and bands were manually detected to quantify the molecular weight and adjusted volume of each band. This enabled comparison of how the bands changed overtime with bleaching. 

## Supplementary Materials

The following are available online at https://www.mdpi.com/2072-6651/11/5/266/s1, Table S1. Change in intensity (adjusted volume) of protein bands from the gel electrophoresis throughout the bleaching process. Quantification was performed in Image Lab 6.0 (BioRad); Figure S1. Determination of protein concentration to achieve approximately 50% lethality for week 0 anemone venom samples (n = 1); Figure S2. Determination of protein concentration to achieve approximately 50% lysis for week 0 anemone venom samples (n = 3); Figure S3. Wavelength of light anemones from each group were exposed to over a period of five months to gradually induce bleaching (**a**) Fluorescent lighting (**b**) LED lighting. Wavelengths were measured using a Lighting Passport Essence (AsenseTek, Taiwan).
