# Supplementary material for: The Ecological Importance of Toxicity: Sea Anemones Maintain Toxic Defence When Bleached
Source: Toxins (Basel). 2019 May 11;11(5):266. doi: 10.3390/toxins11050266 (PMC6563229; doi:10.3390/toxins11050266)
Supplement: Supplementary file 1 [file toxins-11-00266-s001.pdf]

Supplementary Materials: The ecological importance of toxicity: Sea anemones maintain toxic defence when bleached

Cassie M. Hoepner, Catherine A. Abbott and Karen Burke da Silva

Table S1. Change in intensity (adjusted volume) of protein bands from the gel electrophoresis throughout the bleaching process. Quantification was performed in Image Lab 6.0 (BioRad, USA)

	Week	11 kDa	16 kDa	18 kDa	75 kDa
**Anemone 1**	*0*	1,290,678	560,967	0	79,121
	9	1,949,873	434,449	251,766	109,624
	18	96,276	9,727	0	0
**Anemone 2**	*0*	165,217	24,211	0	29,110
	9	203,273	52,256	25,347	56,232
	18	236,785	6,603	0	0

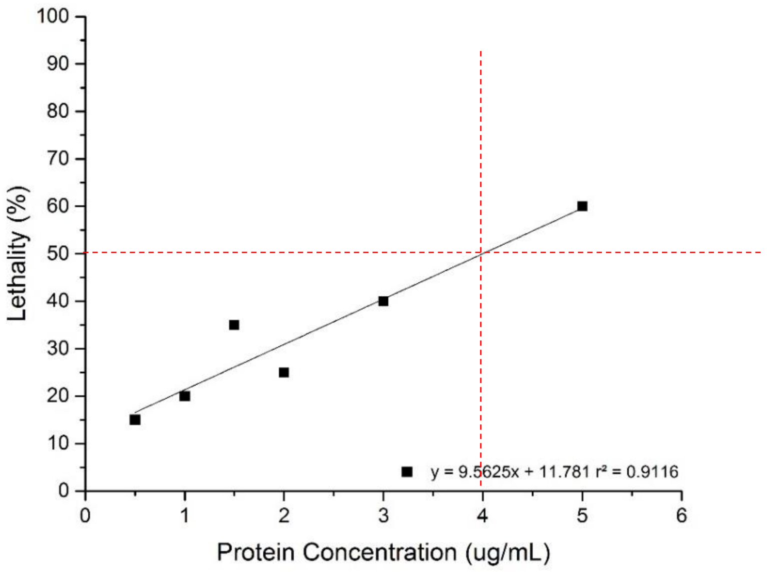

Figure S1. Determination of protein concentration to achieve approximately 50% lethality for week 0 anemone venom samples (n = 1).

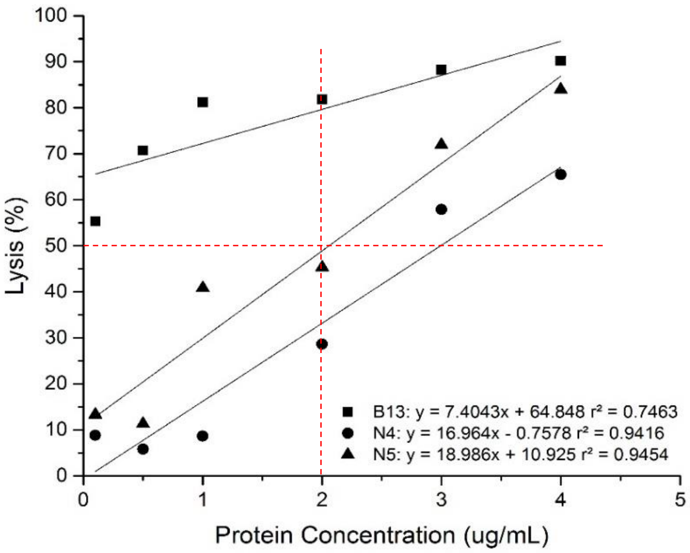

Figure S2. Determination of protein concentration to achieve approximately 50% lysis for week 0 anemone venom samples (n = 3).

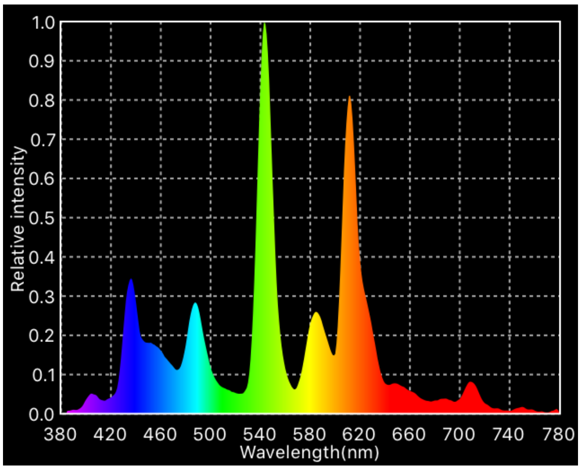

(a)

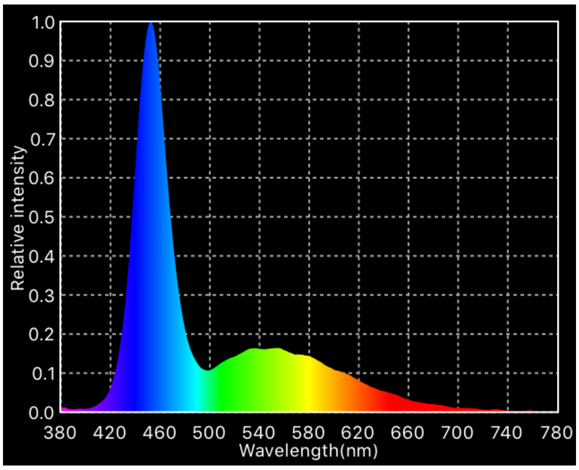

(b)

Figure S3. Wavelength of light anemones from each group were exposed to over a period of 5 months to gradually induce bleaching (**a**) Fluorescent lighting (**b**) LED lighting. Wavelengths were measured using a Lighting Passport Essence (AsenseTek, Taiwan).
